# Supplementary material for: Sorting at embryonic boundaries requires high heterotypic interfacial tension
Source: Nat Commun. 2017 Jul 31;8:157. doi: 10.1038/s41467-017-00146-x (PMC5537356; doi:10.1038/s41467-017-00146-x)
Supplement: Supplementary file 2 — Supplementary Software 1 [file 41467_2017_146_MOESM2_ESM.zip › PottsModel/SrcPottsModel/doc/model/Constants.PathManager.html]

Constants.PathManager


---


|  |  |  |
| --- | --- | --- |
| **Overview** \| **Package** \| **Class** \| **Use** \| **Tree** \| **Deprecated** \| **Index** \| **Help** | |  |
| **PREV CLASS**   **NEXT CLASS** | **FRAMES**    **NO FRAMES**     **All Classes** |  |
| SUMMARY: NESTED \| FIELD \| CONSTR \| METHOD | DETAIL: FIELD \| CONSTR \| METHOD |  |


---


## model Class Constants.PathManager

```
java.lang.Object
  model.Constants.Manager
      model.Constants.PathManager
```

**Enclosing class:**: Constants

---

``` private class Constants.PathManager extends Constants.Manager ```

Manages file paths.

---

| **Field Summary** | |
| --- | --- |
| `private  java.lang.String` | `baseConfigFilename` |
| `private  java.lang.String` | `baseInitialLatticeFilename` |
| `private  java.lang.String` | `baseLatticeDirectory` |
| `private  java.lang.String` | `baseSnapshotDirectory` |
| `private  java.lang.String` | `baseStatsFilename` |
| `private  java.lang.String` | `configFilename` |
| `private  java.lang.String` | `defaultRootDirectory` |
| `private  java.lang.String` | `initialLatticeFilename` |
| `private  java.lang.String` | `latticeDirectory` |
| `private  boolean` | `pathsCreated` |
| `private  java.lang.String` | `rootDirectory` |
| `private  java.lang.String` | `snapshotDirectory` |
| `private  java.lang.String` | `statsFilename` |

**Fields inherited from class model.Constants.Manager**

`prop`


**Constructor Summary**

`Constants.PathManager(java.util.Properties pProperties)`


| **Method Summary** | |
| --- | --- |
| `private  void` | `createPaths()` |
| `private  java.lang.String` | `getLatticeFilename(int mcs)` |
| `private  java.lang.String` | `getSnapshotFilename(int mcs)` |
| `(package private)  void` | `processProperties()` |
| `private  void` | `setInitialLatticeFilename(java.lang.String path)` |
| `private  void` | `setRootDirectory(java.lang.String directory)` |

**Methods inherited from class java.lang.Object**

`clone, equals, finalize, getClass, hashCode, notify, notifyAll, toString, wait, wait, wait`

**Field Detail**

### pathsCreated

```
private boolean pathsCreated
```

---


### snapshotDirectory

```
private java.lang.String snapshotDirectory
```

---


### latticeDirectory

```
private java.lang.String latticeDirectory
```

---


### statsFilename

```
private java.lang.String statsFilename
```

---


### configFilename

```
private java.lang.String configFilename
```

---


### initialLatticeFilename

```
private java.lang.String initialLatticeFilename
```

---


### rootDirectory

```
private java.lang.String rootDirectory
```

---


### defaultRootDirectory

```
private final java.lang.String defaultRootDirectory
```

---


### baseSnapshotDirectory

```
private java.lang.String baseSnapshotDirectory
```

---


### baseLatticeDirectory

```
private java.lang.String baseLatticeDirectory
```

---


### baseStatsFilename

```
private java.lang.String baseStatsFilename
```

---


### baseConfigFilename

```
private java.lang.String baseConfigFilename
```

---


### baseInitialLatticeFilename

```
private java.lang.String baseInitialLatticeFilename
```


**Constructor Detail**

### Constants.PathManager

```
public Constants.PathManager(java.util.Properties pProperties)
```


**Method Detail**

### processProperties

```
void processProperties()
```

:   **Specified by:**: `processProperties` in class `Constants.Manager`

---


### setInitialLatticeFilename

```
private void setInitialLatticeFilename(java.lang.String path)
```

---


### setRootDirectory

```
private void setRootDirectory(java.lang.String directory)
```

---


### getLatticeFilename

```
private java.lang.String getLatticeFilename(int mcs)
```

---


### getSnapshotFilename

```
private java.lang.String getSnapshotFilename(int mcs)
```

---


### createPaths

```
private void createPaths()
```


---


|  |  |  |
| --- | --- | --- |
| **Overview** \| **Package** \| **Class** \| **Use** \| **Tree** \| **Deprecated** \| **Index** \| **Help** | |  |
| **PREV CLASS**   **NEXT CLASS** | **FRAMES**    **NO FRAMES**     **All Classes** |  |
| SUMMARY: NESTED \| FIELD \| CONSTR \| METHOD | DETAIL: FIELD \| CONSTR \| METHOD |  |


---
